# Exploring the Priorities for Home Self-Management Among Patients with Interstitial Lung Disease: A Mixed-Methods Approach Using Q-Methodology

**DOI:** 10.3390/healthcare14142062

**Published:** 2026-07-09

**Authors:** Jiayu Liu, Haibo Ma, Hongyan Lu, Ruijie Gu, Lulu Qi, Yanan Deng, Jianghong Liu

**Affiliations:** 1 School of Nursing, Ningxia Medical University, Yinchuan 750004, China; 20220610246@nxmu.edu.cn (J.L.);; 2 Department of Nursing, General Hospital of Ningxia Medical University, Yinchuan 750004, China

**Keywords:** interstitial lung disease, home-based self-management, patient needs, Q-methodology

## Abstract

**Highlights:**

**What are the main findings?**

- Although often underrecognized and undertreated, interstitial lung disease (ILD) leads to substantial disease burden and severely impairs daily functioning and quality of life for affected patients.
- Patients with ILD experience complex and diverse home self-management needs, covering symptom control, daily adaptation, psychological adjustment, and social support, which directly affect their long-term disease outcomes.

**What are the implications of the main findings?**

- There is an urgent need for healthcare providers, researchers, and related stakeholders to further clarify the priority of home self-management needs in patients with ILD and develop targeted, individualized self-management support strategies.

**Abstract:**

**Aim:** This study aims to analyze the self-management needs of patients with interstitial lung disease, clarify the types and characteristics of their needs, and provide a scientific basis for nursing staff to formulate personalized continuing care plans. **Method:** The Q-methodology was adopted in this study. Semi-structured interviews with 13 patients with ILD and systematic literature retrieval were performed to qualitatively explore and summarize patients’ authentic home self-management needs. The Q-set of representative statement items was further established based on the integrated results of interviews and literature analysis. Subsequently, another 15 patients with ILD were enrolled as the P-set to complete the Q-sort ranking of all statement items. By-person factor analysis was conducted to extract potential self-management demand patterns and refine the typology and core characteristics of patients’ self-management needs. **Result:** Factor analysis identified four common factors, representing four types of home self-management needs in interstitial lung disease patients. These types differ in symptom management, information preferences, and psychological rehabilitation needs, while sharing core common needs. **Conclusions:** Patients with interstitial lung diseases have both common and individual self-management needs, with no universal model. Nurses should develop personalized continuing care plans to improve care precision and adaptability.

## 1. Introduction

Interstitial lung disease (ILD) is a heterogeneous group of diffuse parenchymal lung disorders with complex etiologies, which can be divided into known causes and idiopathic causes. Its core pathological manifestations are alveolar inflammation and/or pulmonary fibrosis. As the disease progresses, the normal structure of the lungs is gradually damaged, and lung function declines continuously, making ILD a major threat to respiratory health worldwide [1,2,3]. ILD presents distinct clinical symptoms, mainly including cough, progressive dyspnea, and decreased exercise tolerance, which markedly impair patients’ quality of life. Epidemiological data reveal that the global prevalence of ILD is increasingly serious. According to the Global Burden of Disease Study, the global prevalence of ILD rose from 0.0617% in 1990 to 0.0816% in 2017 [4]. Meanwhile, post-coronavirus disease 2019 (COVID-19) sequelae have further aggravated the disease burden of ILD. Multiple studies have confirmed that, given the high reinfection rate of severe acute respiratory syndrome coronavirus 2, ILD has become one of the most serious long-term complications following COVID-19 infection [5,6,7,8]. Most patients with severe and critical COVID-19 still suffer from persistent interstitial lung lesions after clinical recovery, which fully demonstrates the strong practical necessity of long-term and standardized management for post-COVID-19 ILD.

Patients with interstitial lung disease (ILD) suffer from symptoms such as dyspnea, fatigue, and anxiety, accompanied by impaired physical function and health-related quality of life. They also encounter difficulties, including economic pressure, social isolation, and social stigma [9,10]. Despite continuous advances in the clinical diagnosis and treatment of ILD, patients show suboptimal performance in home-based self-management. After discharge, most patients are burdened with severe symptoms, insufficient disease-related knowledge, and poor self-management abilities, which compromise the outcomes of home care [11,12]. Currently, there is no curative therapy for ILD. Individualized regimens combining pharmacotherapy, supportive care, and rehabilitation interventions are therefore required to slow disease progression [13]. Meanwhile, patients with ILD demonstrate a strong willingness to actively manage their conditions and have an urgent demand for relevant disease knowledge and management skills. Home-based self-management refers to a set of comprehensive behaviors adopted by patients and caregivers to cope with chronic illnesses and maintain quality of life [13], enabling patients to address physical and psychosocial health challenges [14]. Implementing home-based self-management interventions can also alleviate the current strain on limited medical resources and rising healthcare costs [13,14,15,16]. It has been verified that self-management interventions can improve HRQOL in patients with chronic obstructive pulmonary disease (COPD) and asthma and are recommended by clinical guidelines [17,18,19,20]. Due to ILD’s unique pathology, treatments, and disease progression, current home self-management strategies are not applicable to this population, and no corresponding systematic reviews have been published [21,22,23].

Q-methodology stands out as a purpose-built mixed-methods approach that seamlessly integrates qualitative interpretation with quantitative statistics, specifically designed to systematically excavate individuals’ inner views and preference structures—making it exceptionally well-suited for studies centered on subjective demand exploration. Unlike conventional mixed-methods designs (e.g., separate survey and interview phases), which often struggle to move beyond average scores or fragmented narratives when capturing attitude heterogeneity, Q-methodology transcends these limitations by treating the person rather than the variable as the unit of analysis. Through the process of ranking standardized statements regarding home self-management, this approach can simultaneously quantify shared consensus and distinctly cluster heterogeneous attitudes within the sample, thereby revealing latent typologies of subjective experience [24]. To address the research gap in understanding patients’ unmet home self-management needs, this study used Q-methodology to explore patients’ multiple perspectives on self-management. The ultimate goal is to analyze core demand characteristics and lay an empirical foundation for developing personalized transitional care strategies for patients with ILD.

## 2. Method

### 2.1. Design

This study adopted Q-methodology to explore the cognition and preferences of patients with interstitial lung disease (ILD) regarding home-based self-management. The purposes were to identify patients’ common tendency of home self-management needs, analyze their core self-management demands, and clarify the priorities and deficiencies in home care. Ultimately, this study aims to provide solid empirical evidence for developing scientific, feasible, and individualized transitional care programs tailored for patients with ILD. This study did not require clinical trial registration as it was not within the scope of clinical trials.

### 2.2. Development of Q-Statements

#### 2.2.1. Interview Participants

A purposive sampling method was employed to select 13 patients with interstitial lung disease (ILD) who were treated at the Department of Respiratory and Critical Care Medicine of a tertiary Grade A hospital in Yinchuan, Ningxia Hui Autonomous Region, China, between August and November 2024 as interview participants. Inclusion criteria: (1) met the diagnostic criteria for ILD [3]; (2) aged ≥18 years; (3) Voluntarily signed the informed consent form. Exclusion criteria: (1) patients with other severe cardiac, cerebral, hepatic, or renal diseases; (2) patients with hearing impairment and/or speech dysfunction; (3) patients with mental or cognitive dysfunction.

#### 2.2.2. Generation of Q-Statements

Q statements are a collection of viewpoints related to the research topic, which can be presented in multiple forms, including text, video, and audio [25]. In this study, all Q statements are presented in textual form and jointly developed through two approaches: mining data from semi-structured interviews and extracting content from domestic and foreign literature.

This semi-structured interview process was strictly implemented in accordance with the Consolidated Criteria for Reporting Qualitative Research (COREQ) [26] to ensure the standardization, transparency, and reproducibility of qualitative data collection. Prior to formal interviews, standardized and unified training was conducted for all interviewers. The training covered research purposes, interpretation of the interview outline, communication skills, standardized interviewing rules, privacy protection principles, and emergency response strategies, aiming to standardize the whole interview process and minimize subjective bias. All interviews were carried out in quiet, private, and undisturbed independent consulting rooms or in confidential online environments. Interviews were conducted in Mandarin or local dialects based on respondents’ daily communication habits to fully ensure their comfort and the authenticity of their expressions. The detailed interview guideline is shown in Table 1. A total of 13 patients with interstitial lung disease (ILD) participated in the semi-structured interviews. Focusing on the practical difficulties, subjective feelings, and core needs of patients during home self-management, the interviews aimed to fully collect patients’ authentic and original viewpoints on home self-management. Each interview lasted 10 to 30 min, and a full audio recording was performed with the informed consent of the respondents.

After the interviews, the audio recordings were transcribed verbatim into textual data and preliminarily cleaned and sorted. Redundant oral expressions, repetitive descriptions, and irrelevant content were removed to accurately retain the core original viewpoints of respondents. Interviews and data transcription continued until data saturation was reached and no new viewpoints emerged. Subsequently, thematic analysis was applied to conduct in-depth coding analysis on the sorted interview texts. Two uniformly trained researchers performed independent back-to-back coding to complete the initial coding and theme extraction. Cross-verification was conducted after coding. Discrepant items were resolved through group discussion and rechecking of the original interview data so as to guarantee the objectivity and credibility of the coding results. Ultimately, 13 viewpoint statements reflecting the real home self-management experience of patients with ILD were summarized and extracted from the interview data.

In terms of literature mining, researchers systematically retrieved mainstream domestic and foreign databases, including China National Knowledge Infrastructure (CNKI), Wanfang Data, and PubMed, with the retrieval period limited to the latest decade. Academic papers and clinical guidelines related to home self-management, continuous care, and the health needs of patients with ILD were comprehensively collected. Through item-by-item screening, reading, and summarization, a total of 33 standardized statements concerning the home self-management needs of patients with ILD were extracted from 24 high-quality relevant studies, which provided sufficient theoretical and empirical support for the construction of the Q statement library.

#### 2.2.3. Screening and Optimization

Statements derived from the above sources were collated, while overly professional jargon and redundant content were eliminated. All reserved items were further revised for consistent linguistic standardization. Subsequently, two rounds of modifications were conducted during two research group meetings, with professional review and guidance provided by two nursing experts (including a chief nurse and an associate chief nurse). In accordance with standardized requirements for Q-sort items [27], a total of 35 formal need statements were ultimately finalized. All items were shuffled, randomly numbered, and printed on 35 cards (4 cm × 5 cm) for the subsequent Q-sorting procedure.

### 2.3. Participants

In this study, participants who completed Q-sorting were defined as the P sample, whose inclusion and exclusion criteria were consistent with those of the semi-structured interview participants mentioned above. Maximum variation sampling was adopted to recruit participants. This sampling method can fully reflect the heterogeneity of viewpoints within a small sample, which conforms to the core characteristics of Q-methodology in deeply exploring the subjective perceptions and needs of targeted populations. Participants were patients with interstitial lung disease (ILD) who attended the Department of Respiratory and Critical Care Medicine of a tertiary Grade A hospital in Yinchuan, Ningxia Hui Autonomous Region, from December 2024 to January 2025. A total of 17 patients were recruited for the Q-sort survey, and 15 valid samples were ultimately obtained.

The sample size was strictly determined in accordance with the theoretical specifications and data analysis requirements of Q-methodology. Firstly, Q-methodology is centered on by-person factor analysis, with generally 2 to 5 common factors extracted in relevant studies. Each factor requires support from at least 4 to 6 participants to ensure distinct factor characteristics and stable classification results [28]. Secondly, the ratio of statement items to sample size is critical to analytical reliability, and a ratio of less than 2:1 between Q statements and participants is widely recognized in academic research [29]. Given the 35 Q statements developed in this study, the maximum feasible sample size was calculated to be 17. In addition, the sample size adopted in this study is consistent with the sample sizes of similar Q-methodology studies in the domestic and international nursing fields, indicating that the sample design is sufficiently supported by a theoretical basis and practical evidence.

The reasons for selecting a sample size of 17 are as follows: First, the Q-Methodology adopts by-person factor analysis, aiming to explore the similarities and differences in the home self-management needs of patients with interstitial lung disease. In the Q-Methodology, 2 to 5 factors are usually extracted, and 4 to 6 participants are required to clearly identify each factor [28]. Furthermore, the ratio of Q statements to the number of participants should be less than 2:1. Since 35 Q statements were set in this study, the recommended sample size was no more than 17.

### 2.4. Q-Sorting

**Figure 1:**
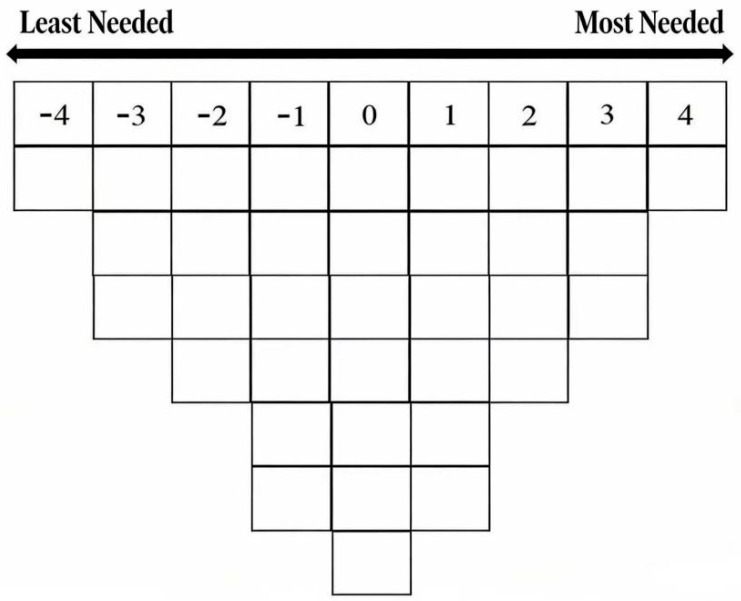
Q-sorting chart.

A 9-point classification method was used to create a quasi-normal distribution table for Q-sorting. Different scores were assigned to each column in descending order based on the degree of need for the viewpoints: the most needed ones were scored “+4”, and the least needed ones were scored “−4”. The scoring method is shown in Figure 1. The 35 cards (Q statements) and the scoring table were presented to the participants, who were invited to read and understand all the cards first. Then, they were asked to place each card in the corresponding cell of the scoring table one by one according to their degree of need for the content on the cards. Researchers explained any confusing sentences to the participants. Participants could adjust the positions of the cards during the sorting process until the quasi-normal distribution table was fully filled. The results included two extreme rankings, namely “the most needed” and “the least needed”. Participants were required to explain their extreme opinions to provide a basis for data analysis.

### 2.5. Ethical Consideration

At the outset, written approvals were obtained from the General Hospital of Ningxia Medical University, Yinchuan, Ningxia Hui Autonomous Region, China (IRB Approval No. KYLL-2022-1128) to conduct the study, and all participants provided written informed consent prior to enrollment.

### 2.6. Data Analysis

Data analysis was performed using PQmethod 2.35 following the person-centered standard logic of Q-methodology. The Q-sorting dataset, consisting of 15 participants (P-sets) and 35 Q-statements, was imported with Q-statements as rows and participants as columns. Data preprocessing was conducted to detect missing values, outliers, and the rationality of Q-sorting distribution to ensure data validity. The analytical procedures are outlined as follows:

(1) Principal component analysis (PCA) for factor extraction: PCA was adopted to extract common factors. The Kaiser criterion (eigenvalue >1) served as the primary screening rule. If no components met this criterion, factors were retained until the cumulative explained variance reached no less than 40% to guarantee stable and interpretable factor structures [27,29].
(2) Varimax orthogonal rotation: Varimax rotation was applied to simplify the factor structure, ensuring each participant loaded highly on only one single independent factor and facilitating the classification of cognitive groups.
(3) Calculation and classification of factor loadings: A larger absolute value of factor loading indicates a stronger correlation between participants and the corresponding factor. With the significance level set at *p* < 0.01, the critical factor loading value was calculated using the formula 2.58/(where N refers to the number of Q statements). Given a total of 35 Q statements in this study, the critical value was determined to be 0.436 [30]. Each factor was required to include at least 2 participants. Samples with cross-loadings assigned to two or more factors simultaneously were excluded from further analysis.
(4) Calculation of Q-statement factor scores and screening of characteristic items: Standardized Z-scores were calculated for all Q-statements. For each factor, the four highest-scoring and four lowest-scoring statements were selected as characteristic items to interpret the core demand tendencies of different participant groups.

## 3. Result

### 3.1. Characteristics of Participants

A total of 13 participants were included in the qualitative interviews, and 15 participants were the P sample (Q-sorting subjects) in this study. The average age of the interviewees was 61 years, including 8 males and 5 females; the average age of the P sample was 60 years, with 2 males and 13 females. Details are shown in Table 2.

### 3.2. Top and Bottom 11 Items of Home Self-Management Demand Scores in Patients with ILD

Following the conventional analytical approach of Q methodology [27], we summarized items with extreme demand scores to clarify patients’ overall attitudes. Among the home self-management needs of patients with interstitial lung disease (ILD), the top 11 items with the highest demand scores were mainly concentrated in symptom management and disease information acquisition, reflecting patients’ urgent need for clinical support to address acute and progressive disease symptoms (Table 3). By comparison, the 11 items with the lowest demand scores mainly concerned lifestyle adjustments and social support services, including lifestyle modification and peer support groups. Patients regarded these needs as less urgent than clinical care (Table 4). These high-scoring items intuitively present the priority ranking of patients’ self-management demands.

### 3.3. Classification of ILD Patients Based on Home Self-Management Needs

Taking eigenvalues >1 as the primary screening standard, factors were retained until the cumulative explained variance reached no less than 40% if no components met the eigenvalue criterion. After Varimax rotation, the four common factors accounted for a cumulative explained variance of 66.708%, suggesting that they reflect most cognitive differences among participants. The absolute factor loading threshold was set at 0.436, and larger absolute loading values meant stronger statistical correlation. These four factors represented four typical viewpoints on home self-management needs among patients with ILD. Detailed eigenvalues, variance contributions, and valid participant numbers of each factor are shown in Table 5.

### 3.4. Types of Home Self-Management Needs Among Patients with Interstitial Lung Disease (ILD)

This study integrated quantitative and qualitative data for comprehensive analysis. PQmethod was used to calculate correlation matrices, conduct principal component analysis, and derive factor loadings and standardized Z-scores of Q-statements, which classified participants into four cognitive groups. Original viewpoints from participant interviews were extracted and matched to their corresponding factors. Taking quantitative grouping results as the analytical framework, original interview narratives were adopted to interpret the subjective motivations behind Q-statement scores. This approach enabled mutual verification between quantitative grouping and qualitative interpretation, addressing the limitation that quantitative results alone cannot explain the formation of respondents’ cognition. Four typical categories of home self-management needs among patients with ILD were ultimately identified, and the specific characteristics of each group are presented as follows:

Symptom Management Need Type: This category included participants P1, P5, P8, P9, P10, and P14. Their home self-management needs mainly focus on symptom control and monitoring. Due to the complex and progressive nature of ILD [4], symptoms including dyspnea and cough greatly damage patients’ physical function and quality of life. Accordingly, these patients attached great importance to effective home-based symptom intervention and had a strong desire to master dynamic symptom monitoring skills and receive individualized pulmonary rehabilitation training.

Psychological Adjustment Need Type: Participants P2, P6, and P7 belonged to this group. Their self-management demands centered on psychological care and disease rehabilitation. Since ILD is irreversible and may lead to a poor prognosis, patients in this group frequently suffer from anxiety, depression, and other psychological problems. Existing studies have confirmed that psychosocial factors are closely linked to disease progression [12]. Therefore, they put forward clear requirements for professional psychological intervention and emotional support during home management.

Basic Protection Need Type: This type covered participants P11, P12, P13, and P15. Their needs revolved around maintaining basic physical health and implementing daily disease prevention. Most of these patients had insufficient understanding of the disease, with poor awareness of home safety protection and deficient self-monitoring ability. These problems may lower treatment adherence and cause adverse health effects. Thus, their core demands lay in satisfying basic physical and safety needs, as well as acquiring systematic disease knowledge and fundamental self-management skills.

Comprehensive Rehabilitation Need Type: Participants P3 and P4 were classified into this category. Their needs targeted the whole process of personalized rehabilitation, covering diverse requirements affected by physical, psychological, and social factors. Most patients in this group had long and complicated disease courses. Apart from basic physical demands, they also had high-level needs in symptom management, disease information acquisition, and social support. The long-term disease burden often results in impaired social roles, heavy economic pressure, and family dysfunction, which means multi-dimensional comprehensive interventions are essential for this population (Table 6).

## 4. Discussion

Currently, the clinical management of interstitial lung disease (ILD) mainly depends on pharmacotherapy to slow disease progression, supplemented by adjunctive measures including balanced nutrition and pulmonary rehabilitation training [31]. Consistent with the progressive and heterogeneous clinical features of ILD, patients with different ILD subtypes exhibit common but differentiated home self-management needs. Physiologically, ILD-induced dyspnea impairs respiratory function and increases systemic energy consumption, necessitating individualized dietary guidance to sustain physical capacity. Compromised immune function and persistent fatigue further raise patients’ demands for standardized home safety management. In terms of symptom regulation, the complex, refractory, and progressive nature of ILD renders most patients lacking systematic scientific knowledge and practical experience in symptom monitoring and control. Accordingly, patients express strong demands for mastering professional symptom surveillance skills and conducting personalized pulmonary rehabilitation exercises to ameliorate respiratory dysfunction and physical discomfort. Regarding disease information acquisition, chronic dyspnea substantially disrupts patients’ daily functioning and occupational participation, resulting in impaired overall quality of life throughout the disease course [32,33]. Therefore, most patients urgently require systematic education on disease etiology, mechanisms of progression, pharmacotherapeutic regimens, treatment efficacy, preventive strategies, and standardized follow-up specifications.

This study found that patients with interstitial lung disease (ILD) show low priority for self-management related to lifestyle modification and social integration. On the surface, most patients have inadequate disease awareness and over-rely on medications, ignoring the long-term benefits of lifestyle intervention, rehabilitation exercise, and social participation. Meanwhile, the heavy economic burden limits their ability to improve living conditions and engage in social activities, reducing their willingness to voice relevant needs. Fundamentally, such disparities in self-management demands result from the combined effects of psychological, cultural, health literacy, socioeconomic, and healthcare system factors. The irreversible and progressive nature of ILD makes patients focus merely on relieving immediate physical symptoms while neglecting long-term health management. Traditional medical perceptions and fatalistic attitudes toward chronic diseases lead patients to underestimate the value of non-pharmacological interventions. Insufficient health literacy further weakens their initiative to implement comprehensive self-management. High medical costs and limited medical insurance coverage leave patients unable to afford lifestyle improvement and social engagement. In addition, domestic clinical management mainly focuses on medication and acute exacerbation treatment, with a shortage of supporting resources, including home-based rehabilitation, psychological counseling, and systematic self-management education, which fail to meet patients’ relevant needs.

The heterogeneous self-management needs identified in this study indicate that one-size-fits-all intervention models are inadequate for the ILD population and that targeted, stratified strategies are clinically essential. In this study, four typical self-management need profiles were summarized among patients with ILD, and tailored intervention approaches are proposed accordingly. For patients with Symptom Management Need Type, a standardized daily symptom monitoring system should be established to guide regular recording of cough frequency, symptom severity, and dyspnea levels. Integrated telemedicine platforms can enable real-time doctor-patient interaction, facilitate dynamic optimization of symptom management protocols, and ensure timely professional intervention [34]. For patients with Psychological Adjustment Need Type, routine standardized psychological assessment is recommended to identify anxiety, depression, and emotional distress. Structured peer support groups and evidence-based relaxation techniques, including progressive muscle relaxation and mindfulness-based stress reduction, should be widely implemented to enhance patients’ psychological resilience and social connectivity [35]. For patients with Basic Protection Need Type, diversified digital health education platforms, such as official WeChat channels and short-video media, should be utilized to popularize standardized self-management knowledge. Individualized home-based counseling is necessary for patients with low health literacy to consolidate basic self-management abilities and home safety awareness [36]. For patients with Comprehensive Rehabilitation Need Type, a multidisciplinary team consisting of pulmonologists, rehabilitation therapists, psychologists, and social workers should formulate comprehensive individualized rehabilitation plans. Meanwhile, integrated social support and medical assistance resources should be accessible to alleviate long-term economic and family functional burdens [37].

This study advances research methods and application scenarios, addressing the limitations of existing studies. Previous research mainly focused on single disease subtypes and adopted unidimensional analytical approaches while neglecting population heterogeneity. To address this gap, we categorized patients into four demand subtypes and refined the characteristics of self-management. Given that the existing literature has rarely explored patients’ needs for lifestyle adjustment and social integration, this study established a multi-dimensional framework to analyze the underlying causes in depth. It also breaks the constraints of universal intervention models by developing subtype-specific intervention strategies with higher clinical applicability. Overall, this research overcomes the limitations of traditional studies, clarifies the influencing mechanisms of patients’ needs, and provides empirical evidence and practical implications for individualized and stratified self-management among patients with interstitial lung disease.

This Q-method study uncovered distinct subjective profiles of home self-management needs among patients with ILD and illustrated the reasoning behind each profile. As an exploratory work, it carries inherent limitations. Q-methodology focuses on interpreting individual subjective viewpoints and cannot generate inferential data available in standard mixed-methods research, such as the population prevalence of each need pattern or its quantitative links to clinical and demographic characteristics. This constraint, however, forms the key contribution of our work. The need typologies we derived are not a final conclusion, but a localized theoretical foundation for future large-scale mixed-method investigations. Subsequent researchers may develop targeted assessment scales based on these profiles and adopt an explanatory sequential mixed-method design. Large-scale surveys can first quantify the prevalence and predictors of each need type, followed by in-depth interviews with representative patient subgroups to unpack how different groups make self-management decisions. This sequential approach builds a full body of evidence, bridging the gap between identifying patient needs and developing personalized intervention plans.

### Limitation

This study has several limitations that should be acknowledged. First, participants were recruited from a single tertiary hospital, which may cause potential selection bias and limit the generalizability of the findings. The sample size was relatively small and did not strictly cover all ILD subtypes evenly, which may fail to fully represent the self-management characteristics of patients with rare interstitial lung disease subtypes [38]. Second, this cross-sectional study only captured patients’ self-management needs at a single time point, unable to reflect dynamic changes in self-management demands alongside disease progression, treatment adjustment, and quality of life variation over the long term. Third, although this study systematically analyzed psychological, cultural, literacy, socioeconomic, and systemic factors influencing self-management priorities, the relevant conclusions were interpretative rather than quantitatively verified. Further structural equation modeling or longitudinal verification is required to confirm the causal relationships between influencing factors and self-management behaviors. Fourth, this study focused exclusively on patients’ subjective self-management needs, without incorporating the perspectives of caregivers, clinicians, and rehabilitation therapists. Multi-stakeholder comparative analysis can further optimize targeted intervention strategies.

## 5. Conclusions

This study adopted Q methodology to explore home self-management needs in patients with interstitial lung disease (ILD) and identified four distinct need profiles. The findings can provide a theoretical basis and practical references for developing targeted, patient-centered interventions in future studies.

## 6. Relevance for Clinical Practice

Based on the findings of this study using Q methodology, respiratory clinicians and multidisciplinary care teams can incorporate the prioritized home self-management needs of patients with interstitial lung disease into routine clinical practice and develop targeted, patient-centered self-management interventions to support effective home care, optimize symptom control, and improve overall disease adaptation and quality of life.

## Figures and Tables

**Table 1:** Interview questions.

Interview Questions
1. After being diagnosed with the disease, how has it affected your life? What burdens has it imposed on your family?
2. How do you conduct disease self-management at home (e.g., diet, exercise, pulmonary rehabilitation, prevention of potential complications)? What channels do you usually use to obtain knowledge related to disease self-management?
3. During the entire process from discharge to home self-management, do you have any needs for disease self-management guidance, professional care guidance, psychological and social support, etc.? Please elaborate.
4. What health information do you most want to learn about? What help do you hope we medical staff can provide for you?

**Table 2:** General characteristics of participants.

Item	Interviewees	P Sample
Age (years, $\bar{x}$ ± s)	61.54 (14.67)	60.33 (8.45)
Gender, *n* (%)		
Male	8 (61.5%)	2 (13.3%)
Female	5 (38.5%)	13 (86.7%)
Educational Level, n (%)		
Primary school and below	10 (76.9%)	13 (86.7%)
Junior high school	2 (15.4%)	1 (6.7%)
Senior high school/Technical secondary school	1 (7.7%)	-
College degree and above	-	1 (6.7%)
Residence, n (%)		
Urban	5 (38.5%)	5 (33.3%)
Rural	8 (61.5%)	10 (66.7%)
Disease Duration, n (%)		
≤5 years	6 (46.2%)	6 (40%)
6~9 years	3 (23.1%)	4 (26.7%)
≥10 years	4 (30.7%)	5 (33.3%)

**Table 3:** The 11 items with the highest scores and their respective scores.

Item	Mean
Guidance on correct pulmonary rehabilitation exercise training	2.13
Guidance on accurate self-monitoring of respiratory system status and function-related physiological indicators such as respiration and blood oxygen saturation	1.60
Understanding the clinical manifestations and management strategies of acute exacerbations of the disease	1.33
Understanding how to prevent complications, e.g., pulmonary infection, respiratory failure, etc.	1.33
Understanding the etiology and disease progression of the illness	1.27
Understanding how to prevent complications, e.g., pulmonary infection, respiratory failure, etc.	1.13
Provision of a healthy diet and nutrition guidance	1.00
Understanding medical information such as follow-up time and follow-up precautions	0.93
Understanding the prognosis and end-stage treatment methods of the disease	0.87
Understanding reliable channels for obtaining health information	0.67
Understanding how to take medications correctly and guidance on identifying and managing adverse drug reactions	0.67

**Table 4:** The 11 items with the lowest scores and their respective scores.

Item	Mean
Establish and join a WeChat patient support group to share disease management experience and provide mutual support	−2.27
Guide the correction of unhealthy lifestyle habits, such as smoking, drinking alcohol, and staying up late	−1.87
Participate in rest and recreational activities	−1.53
Provide confidence for me to return to normal work	−1.47
Receive regular home visits	−1.33
Assist in setting up and guiding the management of a home environment suitable for my condition	−1.27
Understand how to utilize medical resources such as community health centers and township health centers	−1.20
Understand the management methods and daily precautions for abnormal urination and defecation	−1.13
Assist caregivers in receiving appropriate psychological counseling to reduce the negative impact of my illness on their lives	−0.93
Guide the correct implementation of home oxygen therapy and inform me of relevant precautions	−0.53
Assist caregivers in understanding disease care skills and obtaining training in emergency response and management capabilities	−0.53

**Table 5:** Q-analysis variance explanation table.

Component	Eigenvalue	Rotated Variance Explained (%)	Cumulative Variance Explained (%)	Number of Significant Participants
1	4.769	20.384	20.384	6
2	2.082	17.119	37.503	3
3	1.763	16.921	54.424	4
4	1.392	12.285	66.708	2

**Table 6:** Specific items and scores of home self-management needs for each type of patient with interstitial lung disease (ILD).

Type	Items with Higher Scores		Items with Lower Scores	
	Item Content	Mean	Item Content	Mean
Symptom Management Need Type	Guidance on mastering correct coughing and sputum expectoration methods	2.17	Establish and join a WeChat patient support group to share disease management experience and provide mutual support	−2.5
	Guidance on correct pulmonary rehabilitation exercise training	1.83	Provide confidence for me to return to normal work	−2.17
	Guidance on accurate self-monitoring of respiratory system status and function-related physiological indicators, such as respiration and blood oxygen saturation	1.83	Participate in rest and recreational activities	−2.00
	Understanding the etiology and disease progression of the illness	1.83	Guide the correction of unhealthy lifestyle habits, such as smoking, drinking alcohol, and staying up late	−2.00
Psychological Adjustment Need Type	Help alleviate negative emotions such as anxiety and depression	3	Guide the correction of unhealthy lifestyle habits, such as smoking, drinking alcohol, and staying up late	−3.67
	Alleviate the guilt of burdening the family due to illness	3	Provide confidence for me to return to normal work	−3.33
	Guidance on correct pulmonary rehabilitation exercise training	2.33	Establish and join a WeChat patient support group to share disease management experience and provide mutual support	−2.33
	Provide support and encouragement when I am worried and panicked about the disease prognosis	2.33	Assist caregivers in understanding disease care skills and obtaining training in emergency response and management capabilities	−2.33
Basic Protection Need Type	Understanding home safety protection knowledge, e.g., fall prevention, scald prevention, etc.	3.25	Assist caregivers in receiving appropriate psychological counseling to reduce the negative impact of my illness on their lives	−2.5
	Improve sleep quality	2.25	Establish and join a WeChat patient support group to share disease management experience and provide mutual support	−2.25
	Guidance on accurate self-monitoring of respiratory system status and function-related physiological indicators, such as respiration and blood oxygen saturation	2.25	Alleviate the guilt of burdening the family due to illness	−1.75
	Understanding the clinical manifestations and management strategies of acute exacerbations of the disease	2	Provide support and encouragement when I am worried and panicked about the disease prognosis	−1.75
Comprehensive Rehabilitation Need Type	Guidance on correct pulmonary rehabilitation exercise training	3	Receive regular home visits	−3.5
	Understanding the prognosis and end-stage treatment methods of the disease	3	Assist caregivers in understanding disease care skills and obtaining training in emergency response and management capabilities	−3.5
	Understanding social security policy information and obtaining support	2.5	Assist in setting up and guiding the management of a home environment suitable for my condition	−3
	Assist in alleviating respiratory symptoms such as shortness of breath and wheezing	2.5		

## Data Availability

The data presented in this study are available on request from the corresponding author due to privacy and ethical restrictions.
